# Choroid plexus volume enlargement in first-episode antipsychotic-naïve schizophrenia

**DOI:** 10.1038/s41537-023-00424-2

**Published:** 2024-01-02

**Authors:** Jiaxin Zeng, Tianwei Zhang, Biqiu Tang, Siyi Li, Li Yao, Jeffrey R. Bishop, John A. Sweeney, Zhenlin Li, Changjian Qiu, Shi Gu, Qiyong Gong, Wenjing Zhang, Su Lui

**Affiliations:** 1 Department of Radiology, and Functional and Molecular Imaging Key Laboratory of Sichuan Province, West China Hospital of Sichuan University, Chengdu, China; 2 Huaxi MR Research Center (HMRRC), West China Hospital of Sichuan University, Chengdu, China; 3 Research Unit of Psychoradiology, Chinese Academy of Medical Sciences, Chengdu, China; 4 School of Computer Science and Engineering, University of Electronic Science and Technology of China, Chengdu, China; 5 Department of Experimental and Clinical Pharmacology, College of Pharmacy, University of Minnesota, Minneapolis, MN USA; 6 Department of Psychiatry, University of Cincinnati Medical Center, Cincinnati, OH USA; 7 Mental Health Center, West China Hospital of Sichuan University, Chengdu, China

**Keywords:** Biomarkers, Neuroscience

## Abstract

Investigation of the choroid plexus in schizophrenia has seen growing interest due to its role in the interaction between neuroinflammation and brain dysfunction. Most previous studies included treated and long-term ill patients, while antipsychotics and illness course might both affect the choroid plexus. Here, we recruited first-episode antipsychotic-naïve schizophrenia patients, performed high-resolution structural brain scan and manually extracted choroid plexus volume. Choroid plexus volume was compared between patients and healthy controls after controlling for age, sex and intracranial volume. Age and sex effects were examined on choroid plexus volume in patient and healthy control groups respectively. In patients, we also examined the correlation of choroid plexus volume with volume measures of cortical and subcortical gray matter, white matter, lateral ventricular as well as symptom severity and cognitive function. Schizophrenia patients showed significantly enlarged choroid plexus volume compared with healthy controls. Choroid plexus volume was positively correlated with age in only patient group and we found significantly larger choroid plexus volumes in males than females in both patient and healthy control groups, while the sex effects did not differ between groups. Choroid plexus volume was only found correlated with lateral ventricular volume among the brain volume measures. No significant correlation between choroid plexus volume and clinical ratings or cognitive performance was observed. Without potential confounding effects of pharmacotherapy or illness course, our findings indicated the enlargement of choroid plexus in schizophrenia might be an enduring trait for schizophrenia.

## Introduction

Several studies have suggested a role for neuroinflammation in the pathophysiology of schizophrenia, supported by evidence of alterations in immune cells and cytokine levels in both peripheral blood and cerebrospinal fluid (CSF)^1^. Choroid plexus (ChP), the principal source of CSF production and vital structure of the blood-CSF barrier, serves as an immunological barrier regulating the trafficking of immune cells entry into the central nervous system^2^. Recent interest regarding the ChP in schizophrenia has been stimulated by increased understandings of interactions between immune activation and brain dysfunction^3–5^. Transcriptome sequencing of the ChP in schizophrenia has shown predominantly upregulated gene expression related to immune function and inflammation compared with healthy individuals^6^. Conversely, proinflammatory cytokines including tumor necrosis factor α (TNF-α) and interleukin (IL)−1β can inhibit ChP gateway function^7^.

ChP structural abnormalities in schizophrenia were described as early as the 1920s, with reports of morphological changes of the ChP epithelium and vascular endothelium^2^. Computed tomography studies observed an association between ChP calcification and psychotic symptoms^8^. Recently, with the development of neuroimaging and data analysis methods, it is possible to delineate ChP volume directly in vivo in schizophrenia^4,9,10^. Zhou et al. manually delineated ChP and found enlarged ChP volumes in first-episode schizophrenia within two weeks of antipsychotic treatment initiation compared with healthy controls^11^. Lizano et al. used FreeSurfer to automatically segment brain structures and found ChP volume enlargement in treated schizophrenia patients of varying illness duration relative to healthy controls^4^.

While findings have been promising, the potential impact of antipsychotic treatment and longer-term illness course on ChP alterations raises questions of whether this finding is a treatment effect or is directly associated with illness biology. This possibility is suggested by findings that antipsychotics can reduce expression and secretion of inflammatory cytokines including IL-6 and TNF-α in human immune cells, reflected by in vitro study^12^, and that elevated immune levels were normalized after antipsychotic treatment in first-episode psychosis^13,14^. Furthermore, a recent histological study demonstrated increased somal width of epithelial cells in the ChP of unmedicated patients but not in those who had been treated^15^. The effects of illness duration on ChP were also evident in neuroimaging studies. Senay et al. showed significant ChP volume increase in early treated psychosis but not in long-term ill patients using manual delineation of ChP^16^. The study also found a positive correlation between antipsychotic dosage and ChP volumes in early-course psychosis^16^. Thus, while prior studies suggested that ChP volume enlargement was involved in schizophrenia, whether such observation is an illness trait relevant to illness itself or secondary to treatment or illness course factors remains to be determined. Studies of antipsychotic-naïve first-episode schizophrenia (AN-FES) patients may help address this question.

In the present study, we recruited a cohorts of 56 antipsychotic-naïve FES patients and 55 demographically matched healthy controls and manually delineated ChP volume using T1-weighted images, which serves as a gold standard for choroid plexus measurement^17^ and has been widely applied in previous studies^16,18^. We aimed to explore ChP structure alterations at acute illness stage prior to antipsychotic treatment, and to determine whether ChP alterations are related to psychotic symptoms, cognitive deficits or anatomic alterations in other brain regions.

## Methods

### Participants

The study was approved by the Ethics Committee on Biomedical Research of West China Hospital of Sichuan University. Written informed consent was obtained from all participants. Diagnosis of schizophrenia was determined using the Structured Clinical Interview for DSM-IV (SCID) and was confirmed one year later by follow-up evaluation. First episode refers to first manifestation of the disorder meeting the diagnostic symptom and duration requirements according to DSM-IV. Duration of untreated psychosis (DUP) was defined as period from the emergence of first psychotic symptom to initiation of drug treatment as assessed using the Nottingham Onset Schedule^19^, based on information provided by patients, family members, and medical records and the mean DUP of AN-FES was 1.29 months. Psychopathological symptoms were assessed using the Positive and Negative Syndrome Scale (PANSS) and Global Assessment of Functioning (GAF) on the day of MRI scanning^20,21^. The Brief Assessment of Cognition in Schizophrenia (BACS) battery was used to assess cognition dysfunction, with Z scores adjusted for age and sex for statistical analysis^22^. Patients had not received antipsychotic treatment or other psychiatric medications before assessments and MRI scanning, and were then treated with the second-generation antipsychotics.

Healthy individuals were recruited from nearby regions via poster advertisement, and screened using the SCID-Non-Patient Edition to confirm the lifetime absence of psychotic, anxiety and mood disorders. They reported no first-degree relatives with a known history of psychiatric illness based on clinical interviews.

Three patients with noticeable head motion on T1-weighted images and one healthy control exceeding three standard deviation of choroid plexus volume in that group were excluded from the study. At last, a total of 56 AN-FES schizophrenia patients and 55 healthy individuals were included in this study, all aged between 16 and 45 years old. The AN-FES and healthy controls matched in sex (χ^2^ = 1.24, *p* = 0.265) and age (*t* = −0.11, *p* = 0.911, Table 1). The exclusion criteria for all individuals included: (1) systemic or neurological illness, especially illness likely to be associated with immune compromise but also a history of head trauma as assessed by clinical evaluations and review of medical records; (2) taking or have taken pro-/anti-inflammation medications within the past 4 weeks preceding to the MRI scans; (3) pregnancy; and (4) contraindications to MRI scans.

**Table 1:** Demographic characteristics of schizophrenia patients and healthy controls.

	Schizophrenia	Healthy controls	*p*	t or χ
Number	56	55		
Age (Mean ± SD)	24.70 ± 6.53	24.80 ± 2.28	0.911	−0.11
Sex (M/F)	23/33	17/38	0.265	1.24
DUP (Month)	1.29 ± 2.23	NA		
GAF	51.43 ± 15.19	NA		
PANSS				
Positive symptom score	24.82 ± 4.15	NA		
Negative symptom score	22.86 ± 5.23	NA		
General psychopathological symptoms score	47.77 ± 6.88	NA		
Total score	95.45 ± 12.46	NA		
BACS (converted to Z score)				
Verbal Memory	−1.53 ± 1.52	0.89 ± 0.83	<0.001*	−10.22
Digit Sequencing	−0.59 ± 1.42	0.69 ± 0.90	<0.001*	−5.51
Token Motor Task	−0.48 ± 1.16	1.06 ± 0.72	<0.001*	−8.11
Verbal Fluency	−2.35 ± 0.71	−1.33 ± 0.71	<0.001*	−7.19
Symbol Coding	−1.01 ± 1.14	0.95 ± 0.73	<0.001*	−10.50
Tower of London	−0.27 ± 1.30	0.60 ± 0.41	<0.001*	−4.63
Total score	−1.53 ± 1.25	0.71 ± 0.62	<0.001*	−11.3

*P* values reflect case control differences between the diagnostic groups.

*SD* Standard deviation, *M* male, *F* female, *PANSS* positive and negative syndrome scale, *DUP* duration of untreated psychosis, *GAF* global assessment of functioning, *BACS* brief assessment of cognition in schizophrenia, *NA* not applicable.

*Indicates significant group differences, *p* < 0.05.

### MRI data acquisition

MRI data of all individuals were collected on a Siemens Trio 3.0 T MRI scanner equipped with a 32-channel heal coil using the Human Connectome Project (HCP) acquisition protocol^23^. High-resolution T1-weighted images were acquired using a magnetization prepared rapid gradient echo (MPRAGE) sequence using the following acquisition protocol: repetition time (TR) = 2400 ms, echo time (TE) = 2.01 ms, inversion time (TI) = 1000 ms, flip angel = 8°, field of view (FOV) = 256 × 256 mm^2^, voxel size = 0.8 mm isotropic. To ensure the high-quality data, MRI technologists checked the images for head motion and noticeable artifacts when scanning was done. If artifacts were visually apparent, the scan was repeated.

### Choroid plexus delineation

The delineation of choroid plexus was carried out on T1-weighted images manually using ITK-SNAP 4.0 (http://www.itksnap.org/pmwiki/pmwiki.php). Two radiologists (JZ and BT) manually delineated choroid plexus in the lateral ventricles for all the individuals slice by slice primarily on coronal plane without knowing individuals’ information, and editing was conducted on sagittal and axial planes. The volumes of choroid plexus from both hemispheres of lateral ventricular were calculated and combined as the choroid plexus volume. The inter-rater reliability was established between 10 random individual ChP delineations between the two raters and high correlation coefficient was obtained (*r* = 0.95, *p* < 0.001). ChP volume was extracted from all individuals.

### Other brain measures

Brain measures including white matter, cortical and subcortical gray matter as well as lateral ventricular volumes were processed using FreeSurfer Version 6.0 (https://surfer.nmr.mgh.harvard.edu/). The “recon-all” pipelines were used for brain segmentation. Briefly, these steps include motion correction, normalization, automatic segmentation and registration. Finally, estimated total intracranial volume (eTIV), volumes of white matter, lateral ventricles, cortical gray matter, as well as subcortical gray matter including thalamus, caudate, pallidum, putamen, hippocampus and amygdala were extracted as regions of interest for evaluating the specificity of choroid plexus alterations relative to known widely-distributed neuroanatomic alterations in schizophrenia.

### Statistical analysis

#### ChP volume alterations in first-episode antipsychotic-naive schizophrenia

A general linear model was used to compare ChP volumes between diagnostic groups, with age, sex and eTIV being included as covariates. The statistical significance level was set as *p* < 0.05 (two tailed).

#### Age and sex effects on ChP volume

We carried out partial correlation analyses between age and ChP volume using eTIV as a covariate in patient and healthy control groups separately. The difference in correlation between age and ChP volume was compared between groups based on the confidence intervals via the application of the “cocor” package (http://comparingcorrelations.org)^24^.

Since ChP is relevant to multifunctional sex hormones and might be affected by sex, we additionally used general linear model to explore the interaction between sex and group on ChP volume and examined sex effects on ChP volumes in patient and healthy control groups separately, controlling for eTIV and age. False discovery rate (FDR) corrections were carried out for multiple comparison corrections for the above mentioned analyses. The statistical significance threshold was set at *p* < 0.05 with FDR correction (two tailed).

#### Correlation with symptoms and cognition

Partial correlation analyses were applied to explore the relationship between ChP volume and DUP, GAF, PANSS (including positive, negative and general psychopathological symptoms scores and total score) as well as subscales and total score of BACS, controlling for age, sex and eTIV. The statistical significance threshold was set at *p* < 0.05 with FDR correction (two tailed).

#### Correlation with other brain structural measures

To explore whether ChP volume is linked to alterations in widely affected brain structure measures or is a more specific/independent deficit in patients, partial correlation analyses were also applied to explore the relationship between ChP volume and brain volume measures of white matter, lateral ventricles, cortical gray matter and subcortical gray matter including thalamus, caudate, pallidum, putamen, hippocampus and amygdala using age, sex and eTIV as covariates. FDR was used to correct the findings for multiple comparisons and the statistical significance level was set as *p* < 0.05 (two tailed).

## Results

### ChP volume alteration in first-episode antipsychotic-naive schizophrenia

**Fig. 1:**
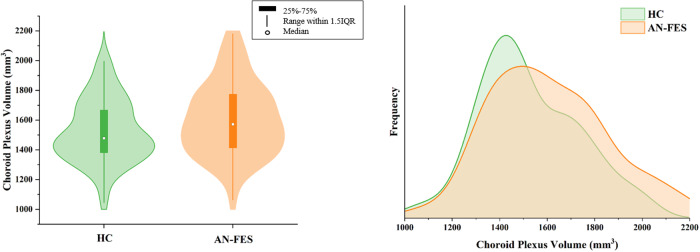
Choroid plexus volume and distribution in schizophrenia patients and healthy controls. AN-FES showed significantly larger choroid plexus volume compared with healthy controls. AN-FES antipsychotic-naïve first-episode schizophrenia, HC healthy controls, IQR interquartile range.

AN-FES (mean ChP volume = 1630.65 mm^3^, 95% confidence interval (CI) = 1549.23–1712.08 mm^3^) showed significantly enlarged ChP volume (*F* = 5.04, *p* = 0.027, partial η^2^ = 0.045, Fig. 1) than matched healthy individuals (mean ChP volume = 1498.21 mm^3^, 95 CI = 1416.03–1580.388 mm^3^).

### Age and sex effects on ChP volume

**Fig. 2:**
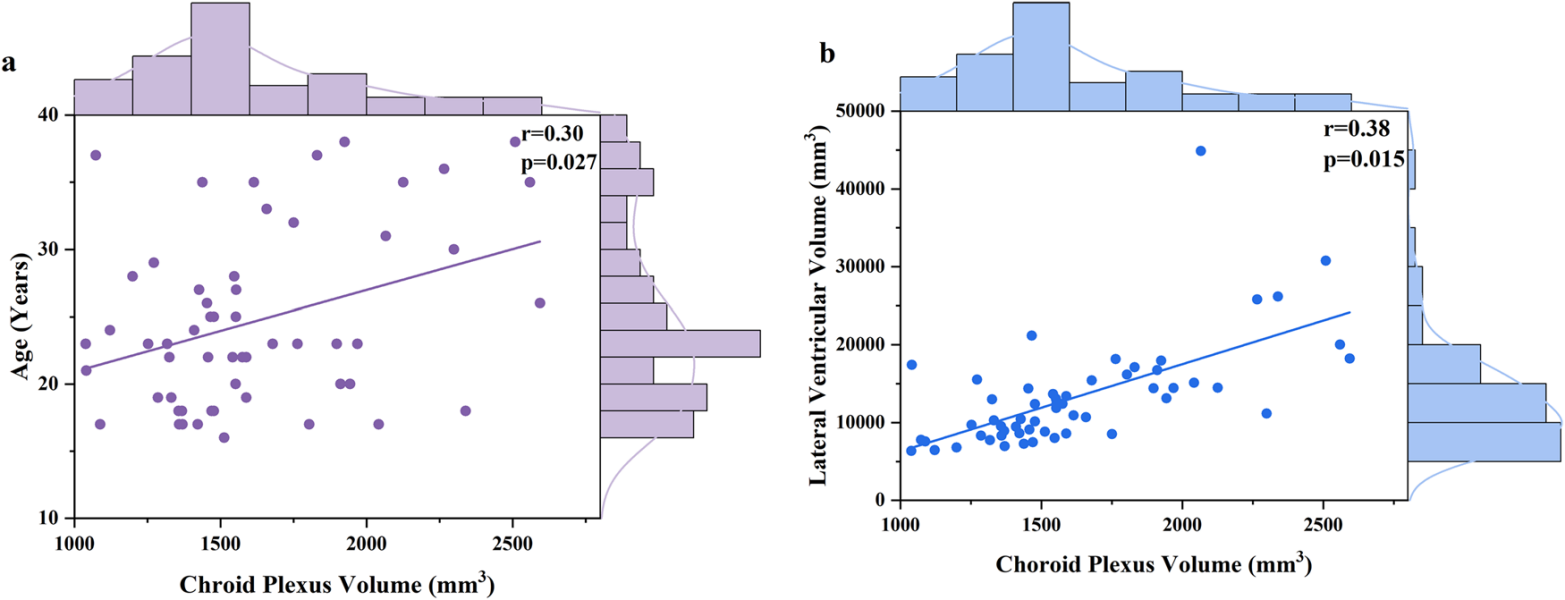
Correlation analyses with choroid plexus volume in schizophrenia patients. **a** Choroid plexus volume was positively correlated with age in patients. **b** Choroid plexus volume was positively correlated with lateral ventricular volume in patients.

Choroid plexus volume enlargement was correlated with older age (*r* = 0.30, *p* = 0.027, Fig. 2) in patients, while we did not find a significant relationship between choroid plexus volume and age in healthy control group (*r* = −0.12, *p* = 0.379). The correlation coefficient of age and choroid plexus volume significantly differed between these two groups (*z* = 2.20, *p* = 0.03).

**Fig. 3:**
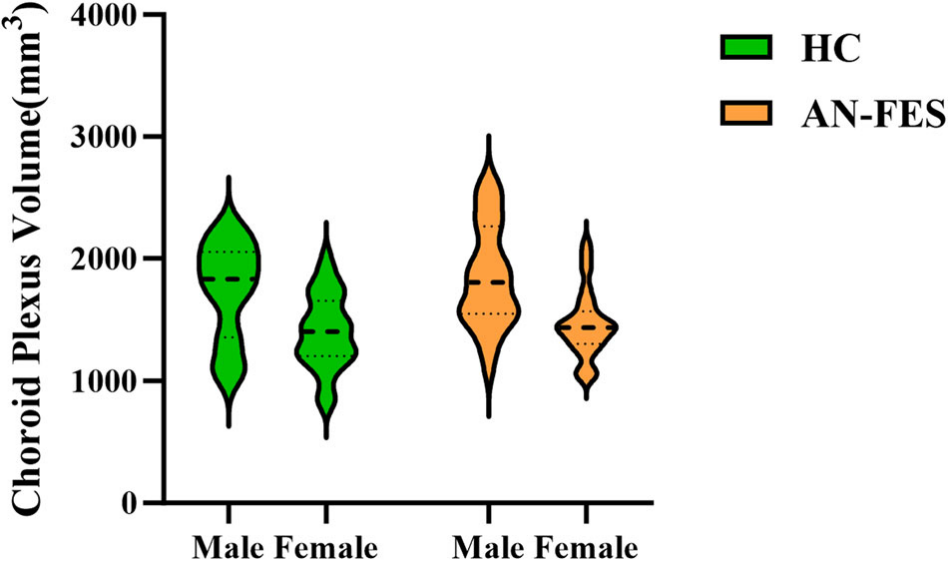
Sex effects on choroid plexus volume in patient and healthy control groups. Males showed significantly larger choroid plexus volume compared with females in both patient and healthy control groups. AN-FES antipsychotic-naïve first-episode schizophrenia, HC healthy controls.

Regarding sex effects on ChP volume across groups, we did not find a significant interaction effect between sex and group (*F* = 0.10, *p* = 0.748), but males showed significantly larger ChP volume compared with females in both schizophrenia patients and healthy controls without controlling for covariates (*F* = 17.65, *p* < 0.001; and *F* = 11.78, *p* = 0.001 respectively, Fig. 3). After adding age and eTIV as covariates, males and females no longer differed in ChP volumes in patient or healthy control groups (*F* = 0.61, *p* = 0.437 and *F* = 4.06, *p* = 0.098 respectively). All *p*-values were corrected with multiple comparisons unless noted otherwise.

### Correlation with symptoms and cognition

The ChP volume was only significantly correlated with DUP (*r* = 0.33, *p* = 0.018) without multiple comparison correction, but the correlation between ChP volume and DUP was no longer significant after correction (*p* = 0.234). ChP volume was not correlated with clinical symptoms rated by GAF or PANSS, or cognitive impairments evaluated with BACS, either with total score or subscales.

### Correlation with other brain structural measures

The ChP volume was positively correlated with lateral ventricle volume (*r* = 0.38, *p* = 0.015, Fig. 2), controlling for age, sex and eTIV. There is no significant correlation between ChP volume and gray matter or white matter measures.

## Discussion

Our results showed enlarged ChP volumes in first-episode schizophrenia patients before antipsychotic drug treatment. The ChP volume enlargement was positively correlated with older age in patients. Interestingly, we found significantly larger ChP volumes in males than females in both patient and healthy control groups, while the sex effects did not differ between groups. We did not observe a significant correlation between ChP volume and clinical assessments of symptom severity or cognition. These findings indicate that ChP volume enlargement in schizophrenia is present early in illness course, and is evident before initiation of antipsychotic treatment.

To our knowledge, this is the first study to document ChP volume enlargement in never treated first-episode schizophrenia patients. The findings of AN-FES patients are important both in supporting the potential importance of choroid plexus enlargement in schizophrenia, but also more broadly in providing inherent alterations in schizophrenia patients. Although previous studies included patients that were either treated or with a long-term illness duration^4,11^, replicated findings have shown ChP volume enlargement in schizophrenia compared to healthy controls. Combining with our findings that ChP volume enlargement is evident in AN-FES and that relations of ChP volume with symptom severity and cognitive deficits in schizophrenia failed to identify distinct clinical features, it may suggest that enlarged ChP volume may be a trait marker of schizophrenia.

The mechanisms of ChP volume enlargement in schizophrenia remain to be fully established. Postmortem studies of schizophrenia, and also of bipolar disorder, have described morphological changes of the ChP, including cellular fat deposition, epithelial hypersecretion and large cystic formations^4^. Moreover, a recent histological study reported increased ChP somal width in untreated schizophrenia patients^15^. Based on these findings, the macroscale enlargement of ChP volume shown from neuroimaging studies such as ours may result from these microscale histology alterations of ChP. These alterations may be related to or result from a persistent neuroinflammation process, as previous studies have established the association between neuroimmune axis and enlarged ChP volume^4^.

Previous studies have shown a positive correlation between age and ChP volume increase across the lifespan in healthy adults^25,26^, with a lower rate of ChP volume hypertrophy before 50 years old^25^. Our failure to identify a correlation between age and ChP volume in healthy controls may relate to narrow age range in the sample. However, enlarged ChP volume was correlated with older age in schizophrenia patients with the same age range of healthy controls. Combining our findings that positive correlation was found between DUP and ChP volume, it raises the possibility that ChP abnormalities are progressive even at the early stage of illness. Our findings raise the importance of studying first-episode schizophrenia patient before it can be influenced by a wide range of illness duration which parallels with age.

Our study showed that ChP structures differ from sex in both control and patient groups without controlling for age and eTIV. Previous study has demonstrated that males have larger ChP volume compare with females in healthy individuals^26^ and in patients with cognitive impairment^10^. The ChP has multifunctional sex hormone targets, containing sex hormone receptors, including estrogen, progesterone and androgen receptors^27^, thus the transcriptome and secretome of ChP and then its macrostructure might be regulated by sex hormones. Evidence has shown that sex hormones can regulate several fundamental biological functions of the ChP, including CSF production and composition, blood-CSF barrier function, and immune surveillance^28^. Estrogens have multiple anti-inflammatory effects and are also known to have modulating effects on neurotransmission^29^. Therefore, the enlarged ChP volume in males compared to females may relate to hormone differences between groups. While these sex effects did not differ between patients and controls, their importance for timing and dosing immunotherapies in schizophrenia patients remains to be evaluated.

Analyses of correlations of choroid plexus enlargement with measures of other brain structures revealed few significant correlations with cortical and subcortical gray matter volumes or white matter volume. This suggests that the cause of choroid plexus enlargement is relatively distinct from the widely distributed pattern of structural brain alterations in schizophrenia. The exception to this pattern was the robust association of choroid plexus enlargement with lateral ventricle volumes. Increased volumes in the lateral ventricles have long been documented in schizophrenia, and previous studies have also reported the positive relationship between ChP and lateral ventricle volumes in psychosis^16,30^. The enlarged ventricles have long been thought to result from neurodegenerative processes in patients showing progressive brain parenchyma atrophy^31^. Our findings raise the possibility that dysfunction of the ChP may be a contributing factor to that well established anatomic alteration in schizophrenia. Last, we note that our failure to establish associations of choroid plexus alteration with volume alterations in brain gray or white matter is inconsistent with some previous studies. For example, larger ChP volume has been associated with smaller amygdala volume^4^. The discrepancy may arise from differing patient characteristics across studies in treatment status and illness duration, as established studies have indicated antipsychotics and illness duration effects on brain white matter and gray matter volume^32^.

The present study has several limitations that need to be taken into consideration. First, our cross-sectional sample limits the exploration of antipsychotics and illness duration effects on ChP volume. However, the AN-FES sample enabled exploring ChP volume at first-episode state before antipsychotics initiation, close to illness onset. Studies of individuals of high risk for schizophrenia may help determine whether this alteration is associated with illness onset or precedes illness onset, and follow-up of AN-FES may establish the stability and trajectory of ChP alterations. Second, novel methods combining deep learning for the automatic segmentation of ChP may be needed to measure ChP volume accurately, which could enhance yield in correlational studies. Finally, our study lacks immune and genetic data, thus cannot establish the link between inflammation dysfunction and ChP alterations directly. Especially, inflammation dysregulation was recently found only in a subgroup but not all patients^5,33^. Therefore, future studies that aim to identify such patient subgroup may help to develop a more effective way to aid the diagnosis and treatment for these patients^34^.

## Conclusions

In conclusion, our study revealed the illness-related ChP volume enlargement in first-episode antipsychotic-naive schizophrenia patients that may serve as a trait measure for schizophrenia patients.

## Data Availability

The analysis methods of this study are available from the corresponding author S.L. upon reasonable request.
